# Low vision services for children in Tanzania

**Published:** 2012

**Authors:** Elizabeth Kishiki, Paul Courtright

**Affiliations:** Childhood Blindness and Low Vision Co-ordinator, Kilimanjaro Centre for Community Ophthalmology; Director, Kilimanjaro Centre for Community Ophthalmology, PO Box 2254, Moshi, Tanzania

**Figure:**
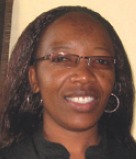
Elizabeth Kishiki

**Figure:**
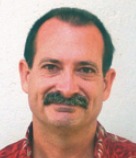
Paul Courtright

The Kilimanjaro Centre for Community Ophthalmology (KCCO) has been involved in a five-year pilot project to improve low vision services for children in Tanzania. Low vision services were limited to a few tertiary hospitals and were accessible to only a few children.

Children with low vision were usually enrolled in schools for the blind, most of them without having received an eye examination or refraction. In these schools, many teachers believe that reading “destroys your vision” and that children with low vision should learn to use Braille; they also believe that all visually impaired children “will lose their sight in the long run.”

To address this, better provision of low vision services and better linkages between education and eye care were needed. To improve provision of low vision services, it was decided to integrate low vision into existing district and regional eye care services, and to train the many optometrists already working at regional or district (population about 1 million) level. One of the key factors in the success of the programme was the appointment of a Childhood Blindness and Low Vision Co-ordinator (Elizabeth Kishiki) to co-ordinate, plan, and teach. Each trained optometrist was also given the lead role in his or her region with a strong report-back mechanism.

**Figure:**
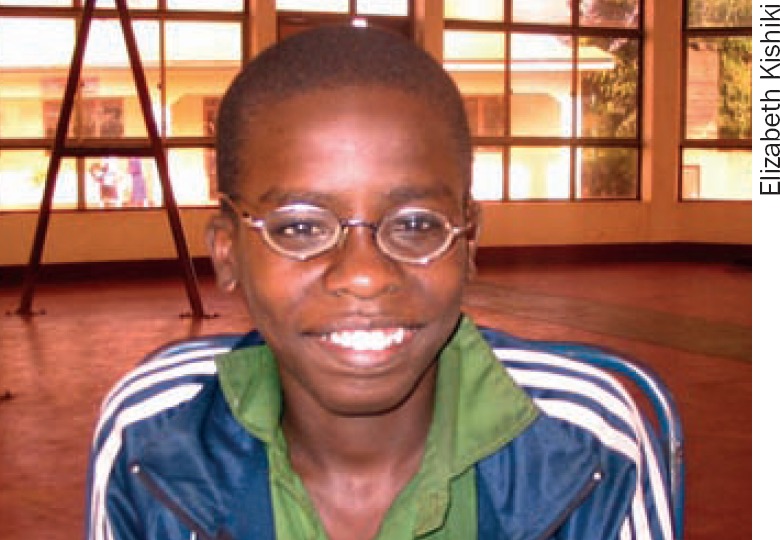
Many children have benefited from the low vision programme. TANZANIA

To improve linkages between education and health services, teachers were trained in the need for regular low vision care and assessment as well as the use of print (rather than Braille), when appropriate. Combining some of the training sessions to include both teachers and optometrists improved co-operation and collaboration. To ensure sustainability, low vision training was included in special education teacher training at the training college in Arusha. Meetings were held to improve collaboration between government, non-governmental organisations, and private stakeholders in both education and eye care. This national consensus had to be translated into action at the regional/district and local level, mainly through training of education and eye care workers.

Follow-up was crucial. SMS reminders were used to ensure that children had clinical follow-ups on an annual basis. The co-ordinator made regular calls to the optometrists, and they would visit schools to troubleshoot as needed.

The training of district special needs education officers, who are responsible for annual budgeting, has in some districts led them to include low vision assessments, optical devices, spectacles, and non-optical devices in their district budgets.

While at the start of the programme, only 13% of children at annexes had been assessed by an eye care professional before admission, after four years, 82% had been assessed and were provided services. Some challenges remain, particularly because so many people and services have to co-operate to make low vision services work, but the benefits have made it worth doing.

*With thanks to Light for the World Netherlands, Seva Canada, and Light for the World Austria*.

